# Influence of Sulfur-Curing Conditions on the Dynamics and Crosslinking of Rubber Networks: A Time-Domain NMR Study

**DOI:** 10.3390/polym14040767

**Published:** 2022-02-16

**Authors:** Francesca Nardelli, Lucia Calucci, Elisa Carignani, Silvia Borsacchi, Mattia Cettolin, Marco Arimondi, Luca Giannini, Marco Geppi, Francesca Martini

**Affiliations:** 1 Dipartimento di Chimica e Chimica Industriale, Università di Pisa, 56124 Pisa, Italy; francesca.nardelli@dcci.unipi.it; 2 Istituto di Chimica dei Composti Organo Metallici, Consiglio Nazionale delle Ricerche, 56124 Pisa, Italy; elisa.carignani@pi.iccom.cnr.it (E.C.); silvia.borsacchi@pi.iccom.cnr.it (S.B.); 3 Centro per l’Integrazione della Strumentazione Scientifica dell’Università di Pisa (CISUP), 56126 Pisa, Italy; 4 Pirelli Tyre SpA, 20126 Milano, Italy; mattia.cettolin@pirelli.com (M.C.); Marco.Arimondi@pirelli.com (M.A.); Luca.Giannini@pirelli.com (L.G.)

**Keywords:** polyisoprene, natural rubber, vulcanization, crosslink density, double quantum NMR, Carr–Purcell–Meiboom–Gill, field cycling NMR

## Abstract

The characterization of the structural and dynamic properties of rubber networks is of fundamental importance in rubber science and technology to design materials with optimized mechanical properties. In this work, natural and isoprene rubber networks obtained by curing at three different temperatures (140, 150, and 170 °C) and three different sulfur contents (1, 2, and 3 phr) in the presence of a 3 phr accelerator were studied using a combination of low-field time-domain NMR (TD-NMR) techniques, including ^1^H multiple-quantum experiments for the measurement of residual dipolar couplings (*D*_res_), the application of the Carr–Purcell–Meiboom–Gill pulse sequence for the measurement of the transverse magnetization decay and the extraction of ^1^H *T*_2_ relaxation times, and the use of field cycling NMR relaxometry for the determination of *T*_1_ relaxation times. The microscopic properties determined by TD-NMR experiments were discussed in comparison with the macroscopic properties obtained using equilibrium swelling, moving die rheometer, and calorimetric techniques. The obtained correlations between NMR observables, crosslink density values, maximum torque values, and glass transition temperatures provided insights into the effects of the vulcanization temperature and accelerator/sulfur ratio on the structure of the polymer networks, as well as on the effects of crosslinking on the segmental dynamics of elastomers. *D*_res_ and *T*_2_ were found to show linear correlations with the crosslink density determined by equilibrium swelling, while *T*_1_ depends on the local dynamics of polymer segments related to the glass transition, which is also affected by chemical modifications of the polymer chains occurring during vulcanization.

## 1. Introduction

The vulcanization of polydiene elastomers by heating with sulfur, accelerators, and other additives is the most widely employed process in the rubber industry to obtain crosslinked materials with thermal, chemical, and mechanical properties suitable for many applications [1,2,3,4], with the production of pneumatic tires being the most famous example. In vulcanization, sulfur reacts with elastomer chains forming crosslinks of different lengths (mainly polysulfidic bonds, but also mono- and disulfidic bonds), pendant chains, and cyclic sulfides. Usually, at the early stage of vulcanization, long polysulfidic links are formed; afterwards, desulfuration and thermal decomposition may occur, which give shorter sulfidic linkages, change the crosslinking degree, and cause main chain modifications. Activators and accelerators are added in order to decrease the vulcanization time, to facilitate the rubber processing, and to improve the vulcanized product properties. In particular, accelerators are able to break the sulfur chains, so that a smaller amount of sulfur is needed for the crosslinking and shorter sulfur fragments are formed in the process, resulting in shorter sulfide bridges in the rubber [5,6]. Accelerated sulfur vulcanizations are classified as conventional (CV, A/S = 0.1 ÷ 0.7), semi-efficient (SEV, A/S = 0.7 ÷ 2.5), and efficient (EV, A/S = 2.5 ÷ 12), depending on the accelerator/sulfur (A/S) ratio, with the amount of both components being given in phr (parts per hundred rubber) [2]. Generally, by increasing the A/S ratio, the efficiency of the vulcanization reaction is improved and a higher number of sulfur bridges of a shorter length are produced [1,4,5,7,8]. Moreover, pendant groups containing the accelerator may form, which constitute elastically ineffective modifications of the elastomer chains; these groups can restrict the mobility of the chain segments and stiffen the chains [4,9,10]. Another fundamental parameter affecting the crosslinking process during vulcanization is temperature, which influences both the vulcanization kinetics and the possible degradation reactions [11,12,13,14]. In particular, it has been found that by increasing the temperature in the accelerated sulfur vulcanization of natural rubber (NR) the crosslink density decreases, decreasing the polysulfidic crosslinks and redistributing the di- and monosulfidic linkages [15,16].

The macroscopic properties of rubbers required for industrial applications are strongly related to the complex structure of the three-dimensional network of crosslinks formed during vulcanization [17,18,19,20,21,22]. This structure is defined by the number and spatial distribution of crosslinks and by their functionality and chemical nature, as well as by the presence of defects (pendant chains, loops, and chain modifications) and entanglements. These properties are in turn related to the vulcanization conditions, although in a quite complex manner [7,8,9,10,13,14,15,16]. Consequently, the investigation of microscopic and macroscopic properties giving access to information on the network structure in relation to vulcanization conditions is extremely important for the optimization of processing and performance of rubbers. 

Information on the average crosslink density (or on the average mass between consecutive crosslinks, *M*_c_) of the network formed by the vulcanization of elastomers can be obtained via different experimental approaches; the most commonly used are equilibrium swelling experiments, mechanical measurements, and proton time-domain NMR (TD-NMR) spectroscopy [1,7,9,10,13,17,18,21,23,24,25,26,27,28,29,30,31,32,33,34,35,36,37]. In several studies, correlations have been drawn between crosslink density measurements obtained by different methods, and origins for discrepancies have been discussed in relation to experimental uncertainties and models adopted in data analysis in the different cases [25,26,28,29,32,37]. Network formation by vulcanization also affects the dynamics of elastomer chains, by imposing constraints to segmental and chain motions and causing strong connectivity between chains. In particular, crosslinking has been found to influence the segmental dynamics connected to the rubber–glass transition (α-relaxation), as shown by dynamic mechanical analysis, dielectric relaxation, and ^1^H field cycling NMR (FC NMR) relaxometry [33,38,39,40,41,42,43].

In the present work, rubbers vulcanized under different conditions were investigated by exploiting both methods that are routinely employed in industrial analyses (i.e., equilibrium swelling, moving die rheometry, MDR, and differential scanning calorimetry, DSC) and TD-NMR experiments, which respectively give access to macroscopic and microscopic properties correlated with the crosslink density. The final aim of the work was to find correlations between NMR observables and the crosslink density or macroscopic properties of the network that depend on this quantity.

Three different ^1^H TD-NMR methods were employed: analysis of transverse relaxation, multiple-quantum (MQ) NMR, and analysis of longitudinal relaxation times measured by FC NMR relaxometry. All NMR observables depend on the modulation of ^1^H-^1^H dipolar couplings by segmental motions. In elastomers, segmental motions are quite fast above *T*_g_, but due to topological constraints imposed by entanglements and crosslinks, they are anisotropic. Therefore, dipolar interactions cannot be fully averaged by segmental dynamics, and weak dipolar couplings remain, which can be eventually averaged over longer temporal and spatial scales by slower polymer motions. On the other hand, isotropic motions can be undergone by terminal dangling chains and small molecules present in the rubbers. In anisotropic systems, ^1^H transverse magnetization decay is mainly induced by residual ^1^H-^1^H dipolar interactions, with effective *T*_2_ time constants that monotonically increase with the degree of mobility. The analysis of transverse magnetization decays acquired using different methods has been extensively used to investigate the phase and dynamic properties of polymeric materials for different applications [44,45,46,47,48]. In the case of rubbers, the presence of solid-like and liquid-like components results in a typical non-exponential decay of the transverse magnetization, which depends on network characteristics [24,26,49,50,51,52], such as the crosslink density and fractions of network chains and dangling chain ends, although the extraction of reliable information on these parameters is not straightforward and strongly depends on both the experimental conditions and model used to fit the decay [53]. Double-quantum (DQ) and more generally MQ NMR techniques allow average residual dipolar couplings (*D*_res_) and their distributions, due to network inhomogeneities, to be quantitatively measured, independently of the time scale of segmental motions. The obtained residual couplings are directly proportional to the crosslink density, although a highly model-dependent procedure is required to extract *M*_c_ from *D*_res_ values [25,27,28,29,37]. FC NMR relaxometry has been seldom employed to measure ^1^H longitudinal relaxation times, *T*_1_ (or rates *R*_1_ = 1/*T*_1_), over a broad range of Larmor frequencies (*ν* = 0.01–40 MHz) on vulcanized elastomers and to extract information on segmental (also referred to as “glassy”) and polymer dynamics [39,40,41,42]. The relaxation rate was found to increase with the crosslink density, as also revealed by measurements at a single frequency [28]. In a recent work, a procedure exploiting the frequency–temperature superposition (FTS) principle and the construction of master curves of the FC NMR susceptibility, *χ*″(ω) = ω*R*_1_(ω) with ω = 2π*ν*, allowed the correlation times for segmental dynamics to be determined as a function of temperature for polyisoprene, polybutadiene, and poly(styrene-co-butadiene) rubbers before and after vulcanization [42]. The correlation times were longer for the sulfur-cured elastomers and increased by increasing the crosslink density, accounting for the observed differences in *R*_1_.

The above discussed TD-NMR methods are exploited here to easily determine extractable parameters on samples prepared from natural rubber (NR) or polyisoprene rubber (IR) by vulcanization with different amounts of sulfur (1, 2, and 3 phr) and a 3 phr accelerator (N-tert-butyl-2-benzothiazole sulfenamide, TBBS) at three different temperatures (140, 150, and 170 °C). The A/S ratios are, therefore, typical of EV (systems with 1 phr sulfur) and SEV (systems with 2 and 3 phr sulfur). Correlations are drawn between *T*_2_, *D*_res_, and *T*_1_ values and macroscopic parameters (crosslink density from equilibrium swelling and *T*_g_) and discussed in reference to vulcanization conditions. Information on the effects of different structural and dynamic features of the polymer networks on the investigated NMR observables is obtained, which could be useful in extending the application of TD-NMR methods as routine characterization techniques for rubber materials. 

## 2. Materials and Methods

### 2.1. Sample Preparation 

All samples were provided by Pirelli Tyre SpA (Milano, Italy). For the preparation of crosslinked rubbers, IR (*cis*-1,4-polyisoprene, ≥96% cis, *M*_w_ = 1.49 × 10^6^ g/mol, and *M*_n_ = 8.41 × 10^5^ g/mol, HC Neftekhimik Nizhnekamsk, Russia) or NR (natural rubber, Standard Indonesian Rubber SIR20) was mixed with 3 phr ZnO (A-ESSE S.p.A., Italy), 2 phr stearic acid (OLEON N.V., Belgium), 2 phr N-(1,3-dimethylbutyl)-N′-phenyl-p-phenylenediamine (6PPD, Eastman Chemical Company, Kingsport, TN, USA) as an antioxidant, 3 phr N-tert-butyl-2-benzothiazole sulfenamide (TBBS, General Quìmica S.A.U., Spain) as an accelerator, and 1, 2, or 3 phr sulfur (S, Zolfindustria Srl, Novara, Italy). The obtained blends were vulcanized into sheets of 1 mm thickness at the curing temperatures (*T*_vulc_) of 140, 150, and 170 °C. Samples vulcanized at the maximum degree of crosslinking were considered for NMR measurements. The codes IR_*T*_vulc_ and NR_*T*_vulc_ are used throughout the work to indicate the series of IR and NR samples vulcanized at *T*_vulc_. When needed, sulfur contents in phr (x) are specified using the codes IR_*T*_vulc__Sx and NR_ *T*_vulc__Sx.

### 2.2. Equilibrium Swelling, MDR, and DSC Experiments 

The crosslink density of the vulcanized rubbers was determined by equilibrium swelling measurements performed in toluene at 20 °C for 72 h, using the Flory–Rehner equation [17]. The values used for the molar volume of toluene and for the Flory–Huggins interaction parameter were 106.28 cm^3^ mol^−1^ and 0.42, respectively. Experiments were performed also on samples treated with the thiol-amine reagent, which breaks polysulfidic links, following established literature procedures [17]. 

Curing profiles were measured with a Moving Die Rheometer MDR (RPA 2000, Alpha Technologies, London, UK). The optimum curing was achieved under the following conditions: ±1° oscillation angle, 4.3 bar pressure and (i) 140 °C for 90–120 min, (ii) 150 °C for 60–90 min, and (iii) 170 °C for 30 min running time. The reported *M*_h_ torque values correspond to the absolute maximum in the MRD curves.

Glass transition temperatures (*T*_g_) were determined by differential scanning calorimetry (DSC) using a DSC Mettler-Toledo 822e instrument (Mettler-Toledo S.p.A., Italy). Thermal cycles between 183 and 323 K were performed and the cooling/heating rate was 10 K/min. *T*_g_ was determined as the intersection point of the two tangents to the DSC curve at the endothermic step. 

### 2.3. NMR Experiments 

^1^H MQ and Carr–Purcell–Meiboom–Gill (CPMG) experiments were carried out on a Bruker Avance Neo spectrometer (Bruker Italia S.r.l., Milano, Italy) working at the ^1^H Larmor frequency of 500.13 MHz, equipped with a 4 mm CP-MAS probe head. Each sample was loaded into a 4 mm rotor after being cut into small pieces. All measurements were performed under static conditions at 25 °C, using a 90° pulse of 2.8 μs and a recycle delay of 5–6 s, accumulating 16 and 32 scans for MQ and CPMG experiments, respectively. A time incremented 1-cycle version of the improved MQ Baum–Pines pulse sequence, including a CYCLOPS phase cycling scheme, was used [29,54]. The intensity of the DQ (*I*_DQ_) and reference (*I*_ref_) signals was measured from the average intensity of the first datapoints (from 2 to 40 μs) of the free induction decay signal obtained for each DQ evolution time (*τ*_DQ_). Here, 48 different *τ*_DQ_ values ranging from 0.1 to 100 ms were used to construct each build-up curve. The alternating-phase CPMG scheme described in [55] (pulse scheme: (π/2)_x_ − (*τ*_echo_ − (π)_y_ − *τ*_echo_ − *acq.* − *τ*_echo_ − (π)_-y_ − *τ*_echo_ − *acq.* − *τ*_echo_ − (π)_-y_ − *τ*_echo_ − *acq.* − *τ*_echo_ − (π)_y_ − *τ*_echo_ − *acq.*)_n_) was applied in order to measure transverse magnetization decay curves without spin-locking effects. For each experiment, 2000 echoes were acquired using *τ*_echo_ = 50 μs.

^1^H longitudinal relaxation times (*T*_1_) were measured at 30 °C at Larmor frequencies from 0.01 to 35 MHz using a Spinmaster FFC-2000 relaxometer (Stelar S.r.l., Mede, Italy). For the experiments, the prepolarizing and non-prepolarizing pulse sequences [56,57] were applied below and above 12 MHz, respectively. The polarizing frequency was set at 25 MHz and the detection frequency at 16.3 MHz. The 90° pulse duration was 9.8 μs and the switching time was 3 ms. A single scan was acquired, using at least 16 values of the variable delay to build the magnetization curves. For each experiment, all other parameters were optimized. All ^1^H magnetization curves showed a monoexponential trend as a function of time within the experimental error. Errors on *R*_1_ were always lower than 2%. All measurements were performed using 10 mm NMR glass tubes. The sample temperature was controlled within ±0.1 °C by a Stelar VTC90 variable temperature unit.

### 2.4. Analysis of the DQ Build-Up Curves

The analysis of the DQ build-up curves was performed on the normalized DQ intensity (*I*_nDQ_):

(1) $$I_{\mathrm{nDQ}}\left(\tau_{\mathrm{DQ}}\right)=\frac{I_{\mathrm{DQ}}\left(\tau_{\mathrm{DQ}}\right)}{I_{\mathrm{DQ}}\left(\tau_{\mathrm{DQ}}\right)+I_{\mathrm{ref}}\left(\tau_{\mathrm{DQ}}\right)-Be^{-\frac{2\tau_{\mathrm{DQ}}}{T_{2B}{}^{*}}}-Ce^{-\frac{2\tau_{\mathrm{DQ}}}{T_{2C}{}^{*}}}}$$

The $Be^{-\frac{2\tau_{\mathrm{DQ}}}{T_{2B}{}^{*}}}$ and $Ce^{-\frac{2\tau_{\mathrm{DQ}}}{T_{2C}{}^{*}}}$ functions can be ascribed to the long-term decays associated with dangling chains and low molecular weight sol components in the rubbers. Since their contribution is contained in *I*_ref_ but not in *I*_DQ_, these components need to be subtracted from *I*_ref_ before proceeding with the correct normalization of the *I*_DQ_ curves. $Be^{-\frac{2\tau_{\mathrm{DQ}}}{T_{2B}{}^{*}}}$ and $Ce^{-\frac{2\tau_{\mathrm{DQ}}}{T_{2C}{}^{*}}}$ were obtained by fitting the long time dependence of *I*_ref_ − *I*_DQ_, similarly to what was reported in [58]. 

Average residual dipolar coupling (*D*_res_) values were obtained by fitting the short time dependence of the experimental *I*_nDQ_(*τ*_DQ_) build-up curve (up to *I*_nDQ_ = 0.45, [29]) to the analytical function:

(2) $$I_{\mathrm{nDQ}}\left(\tau_{\mathrm{DQ}}\right)=\frac{1}{2}\left(1-e^{-\frac{2}{5}\;{\left(\frac{D_{\mathrm{res}}}{2\pi }\;\tau_{\mathrm{DQ}}\right)}^{2}}\right)$$

### 2.5. Analysis of the ^1^H CPMG NMR Relaxation Curves

^1^H transverse relaxation curves obtained from CPMG experiments were analyzed using a discrete approach, by fitting them to a linear combination of one Weibullian function and one exponential function, each characterized by a weight (*C*_weib_, *C*_exp_) and a *T*_2_ value (*T*_2,weib_, *T*_2,exp_), as reported in Equation (3):

(3) $$I_{\mathrm{CPMG}}\left(t\right)=C_{\mathrm{weib}}\;e^{-{\left(\frac{t}{T_{2,\;\mathrm{weib}}}\right)}^{\beta }}+C_{\exp}\;e^{-\frac{t}{T_{2,\;\exp}}}$$

The Weibullian function was used to describe the decay associated to protons in the network chains fluctuating between constraints; the exponential function was used to reproduce the transverse magnetization decay of protons in less-constrained dangling chains and liquid-like sol components [59,60,61].

The initial slope of the transverse relaxation decays was also estimated by performing a linear fitting of the first ten datapoints of the curves (up to 1 ms).

### 2.6. Analysis of the ^1^H FC NMRD Curves

^1^H NMR dispersion (NMRD) curves were obtained by plotting *R*_1_ as a function of the Larmor frequency. ^1^H NMRD curves were converted to a (non-normalized) susceptibility representation *χ*″(ω) = ω*R*_1_(ω) [62]. The *χ*″(ω) curves present a maximum, usually observed at high frequencies and low temperatures in the case of elastomers, when the condition ω*τ*_s_ ≅ 1 is fulfilled, where *τ*_s_ is the characteristic time of the segmental motions within the Kuhn segments related to glassy dynamics. Under the assumption that the FTS principle holds true [63,64], master curves of *χ*″(ω*τ*_s_) can be obtained by shifting curves recorded at different temperatures along their frequency axis until they overlap. The values of *τ*_s_ can be determined directly from the condition ω*τ*_s_ ≅ 1 for the temperatures at which the *χ*″(ω) maximum is visible and from the shift factors for the other temperatures. 

Using a similar procedure, in this work the *χ*″(ω) curves of the vulcanized samples were shifted along the frequency axis until they overlapped the *χ*″(ω) curve of the corresponding unvulcanized sample, taken as a reference, to obtain *χ*″(ω*k*) master curves, where *k* is the shift factor (*k* = 1 for the reference sample). Although this procedure does not allow a direct determination of *τ*_s_ values, it provides values of *k*, which are proportional to *τ*_s_ through the unknown proportionality constant α, which is equal for all samples based on the same elastomer. As shown in [42], at a given temperature crosslinking has the effect of slowing down glassy dynamics, so that *τ*_s_ progressively becomes longer by increasing the crosslink density; the same trend is expected for the *k* factor.

Based on a reworked version of the Vogel−Fulcher−Tammann equation reported by Blochowicz et al. [65], polymers with the same pre-exponential factor, *τ*_0_, and fragility index, *m*, display the same behavior if Log*τ*_s_ is plotted as a function of (*T*/*T*_g_ − 1) [42]:

(4) $$\mathrm{Log}\left(\frac{\tau_{s}\left(T\right)}{\tau_{0}}\right)=\frac{{\mathrm{Log}}^{2}\left(\frac{\tau_{s}\left(T_{g}\right)}{\tau_{0}}\right)}{m\left(\frac{T}{T_{g}}-1\right)+\mathrm{Log}\left(\frac{\tau_{s}\left(T_{g}\right)}{\tau_{0}}\right)}$$

Equation (4) can also be written as a function of *k* and α:

(5) $$\mathrm{Log}\left(\frac{k\left(T\right)}{\alpha \tau_{0}}\right)=\frac{{\mathrm{Log}}^{2}\left(\frac{\tau_{s}\left(T_{g}\right)}{\tau_{0}}\right)}{m\left(\frac{T}{T_{g}}-1\right)+\mathrm{Log}\left(\frac{\tau_{s}\left(T_{g}\right)}{\tau_{0}}\right)}$$

Therefore, if we assume that *τ*_0_ and *m* do not change, all samples obtained using the same polymer matrix should display the same trend also if Log*k* is plotted as a function of (*T*/*T*_g_ − 1).

## 3. Results and Discussion

### 3.1. Characterization by Equilibrium Swelling, MDR, and DSC

**Figure 1:**
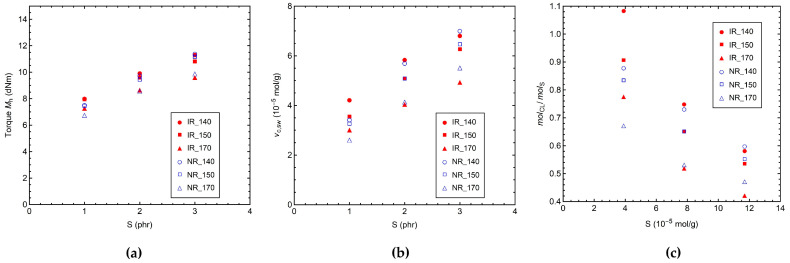
(**a**) Maximum torque (*M*_h_) values from MDR measurements and (**b**) crosslink density values from equilibrium swelling experiments (*ν*_c,sw_) vs. sulfur content (S) in phr. (**c**) Molar ratio (*mol*_CL_/*mol*_S_) values between the total number of crosslinks and the initial amount of sulfur S_8_ plotted vs. the initial molar amount of S_8_ per gram of rubber (S, 10^−5^ mol/g). Values of *mol*_CL_/*mol*_S_ < 1 indicate the presence of unreacted sulfur or the occurrence of side reactions, which do not lead to the formation of elastically active crosslinks.

In Figure 1, the values of the maximum torque (*M*_h_) obtained by MDR measurements and of the crosslink density determined by equilibrium swelling experiments are reported as a function of the initial sulfur content for the IR and NR samples vulcanized at 140, 150, and 170 °C. At each vulcanization temperature (*T*_vulc_), *M*_h_ progressively increases by increasing the sulfur content (Figure 1a), while it decreases with the increase in curing temperature, especially on passing from 150 to 170 °C. The same behavior can be observed for the crosslink density determined by equilibrium swelling (*ν*_c,sw_), as shown in Figure 1b. Moreover, a linear dependence of *M*_h_ on *ν*_c,sw_ can be found (Figure S1), ascribable to the increase in viscosity of the polymer network. In fact, a direct proportionality between *M*_h_ and crosslink density is documented in the literature [8,36]. It is worth noting that since the *ν*_c,sw_ values are in the range 2–8 × 10^−5^ mol/g, our samples can be considered as moderately crosslinked. The decrease in *ν*_c,sw_ by increasing *T*_vulc_ can be explained with a reduction of the vulcanization efficiency, as clearly shown in Figure 1c. Indeed, at elevated curing temperatures, it is more likely that sulfur and sulfurating complexes undergo various uncontrolled side reactions at the expenses of the formation of elastically effective crosslinks. Possible side reactions lead to modifications of the polymer chains, including the formation of cyclic sulfidic structures and unreactive sulfidic accelerator-terminated pendant groups, as well as to the production of zinc sulfide [15,16,66]. Moreover, the curing temperature can also influence the amount and distribution of different crosslink structures in the polymer networks. 

**Figure 2:**
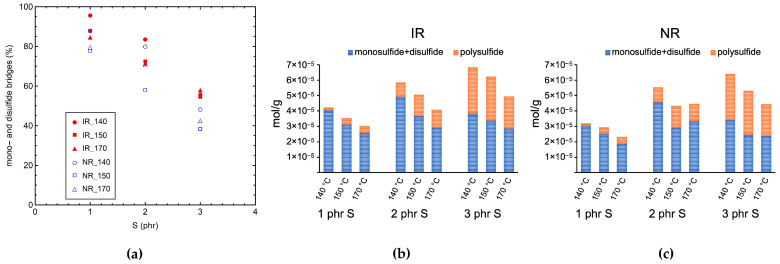
(**a**) Percentage of mono- and disulfide bridges obtained by thiol-amine analysis vs. sulfur content (S). Crosslink density values from equilibrium swelling experiments and types of crosslinks formed in the (**b**) IR and (**c**) NR samples vulcanized at the indicated temperatures and sulfur contents.

As shown in Figure 2, at each *T*_vulc_, the percentage of mono- and disulfide bridges progressively decreases by increasing the sulfur content, probably due to the concomitant decrease in A/S ratio from 3 (EV system) to 1 (SEV system), which favors the formation of polysulfide bridges [5,10,30]. Different trends can be obtained as a function of the curing temperature for samples with different sulfur contents. In particular, a decrease in the percentage of mono- and disulfide bridges occurs for samples containing 1 phr sulfur by increasing the vulcanization temperature, which is ascribable to the faster vulcanization kinetics and shorter curing times, which make the conversion of polysulfide bridges (formed at the initial stages of vulcanization) into more stable mono- and disulfide bridges less efficient [13,14]. This effect is more pronounced when passing from 140 to 150 °C. For IR samples, at 2 phr sulfur the percentage of polysulfide bridges increases from *T*_vulc_ = 140 °C to *T*_vulc_ = 150 °C, and then it remains constant until 170 °C. At 3 phr sulfur, the trend of the percentage of polysulfide bridges with *T*_vulc_ shows a slight maximum at 150 °C. On the other hand, for NR samples, the percentage of polysulfide bridges shows a maximum at 150 °C at both 2 and 3 phr sulfur. The observed trends could be related to the decrease in vulcanization efficiency observed at high *T*_vulc_ and low A/S (Figure 1c), which causes a lower amount of sulfur to be available for the formation of crosslinks. An analogous behavior of the relative amount of polysulfide bridges by increasing the vulcanization temperature was also reported for NR by other authors [14,15,16] and ascribed to a combined effect of vulcanization kinetics and efficiency.

**Figure 3:**
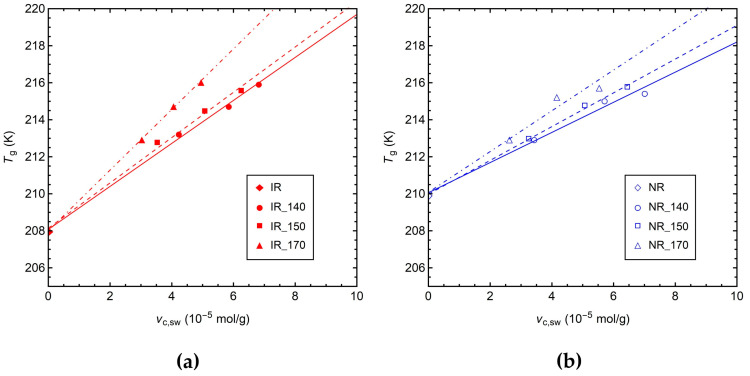
*T*_g_ values measured by DSC vs. *ν*_c,sw_ for (**a**) IR and (**b**) NR samples. Solid, dashed, and dash-dotted lines represent the linear fits to the data for the IR(NR)_140, IR(NR)_150, and IR(NR)_170 series, respectively.

In Figure 3, the values of *T*_g_ measured by DSC are reported vs. *ν*_c,sw_ for all the IR (Figure 3a) and NR (Figure 3b) samples. The values of *T*_g_ of the crosslinked IR and NR samples are higher than those of the corresponding uncured rubbers. In particular, for samples cured at the same temperature, a linear increase in *T*_g_ by increasing the crosslink density can be observed. A linear relationship between the *T*_g_ and crosslink density at a low degree of crosslinking is predicted by theoretical models [18,67] and has been experimentally demonstrated for several crosslinked polymers [9,10,18,42,68]. The increase in *T*_g_ upon increasing crosslinking can be explained by considering that the formation of covalent bonds between polymer chains results in a more compact polymer network, whereby the segmental motions of the chains are significantly hindered and require a higher amount of thermal energy to be activated. Interestingly, samples cured at 170 °C are characterized by higher values of *T*_g_ and by a steeper slope of the linear dependence of *T*_g_ vs. *ν*_c,sw_ compared to samples cured at 140 and 150 °C. In particular, the obtained slopes are 1.16, 1.22, and 1.64 K/(10^−5^ mol/g) for the IR_140, IR_150, and IR_170 series, respectively; and 0.81, 0.91, and 1.10 K/(10^−5^ mol/g) for the NR_140, NR_150, and NR_170 series, respectively. This result could be related to the presence of chemical modifications of the polymer chain, which as discussed above occur in larger amounts at high vulcanization temperatures. Indeed, such modifications, for instance the attachment of cyclic sulfide structures and bulky sulfur or accelerator pendant groups, restrict the mobility of the polymer chains, further contributing to the increase in *T*_g_ (the so-called “copolymer” effect) along with the formation of crosslinks. Similar linear dependences on crosslink density for samples cured at 140 and 150 °C suggest that the decrease in crosslinking efficiency observed at 150 °C (Figure 1b,c) must be mainly ascribed to the presence of unreacted curatives or to the formation of zinc sulfide rather than to the occurrence of chemical chain modifications. 

### 3.2. Characterization by ^1^H TD-NMR 

#### 3.2.1. ^1^H MQ NMR Experiments

^1^H MQ NMR represents one of the most quantitative methods used to determine the average values of *D*_res_ [29,54]. In crosslinked rubbers, *D*_res_ is the result of the incomplete motional average of the ^1^H dipolar interactions due to the presence of chemical crosslinks and physical entanglements, which constrain the motions of polymer segments and induce the persistence of a residual local order even at temperatures far above the glass transition. 

**Figure 4:**
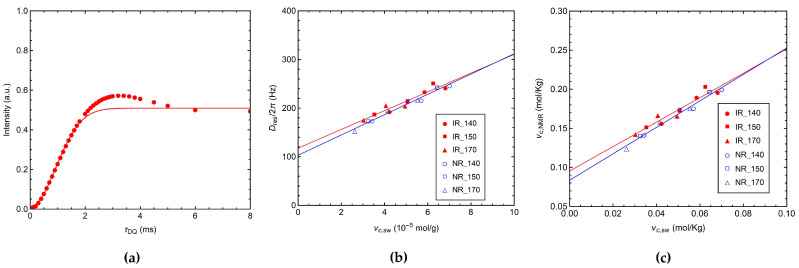
(**a**) *I*_nDQ_ build-up curve of IR_140_S1; the solid line represents the fit obtained using Equation (2). (**b**) *D*_res_ and (**c**) *ν*_c,NMR_ values obtained by MQ experiments vs. *ν*_c,sw_. Solid lines indicate linear fits to the data for IR (red) and NR (blue) series.

The values of *D*_res_ for all vulcanized IR and NR samples were obtained by analyzing the initial build-up of the normalized DQ signal (*I*_nDQ_) using Equation (2), as described in Section 2.4. The fitting of the *I*_nDQ_ build-up curve of IR_140_S1 is shown as an example in Figure 4a. 

In all cases, a linear dependence of *D*_res_ on *ν*_c,sw_ was found (Figure 4b), with slopes of 19 and 21 Hz/(10^−5^ mol/g) and intercepts of 120 and 103 Hz for IR and NR samples, respectively. The linear correlations found for all the crosslinked samples indicate that neither *D*_res_ nor *ν*_c,sw_ are affected by the chemical modifications of the polymer chains, which were found to mainly occur at high vulcanization temperatures. These results are in line with those reported in the literature for different kinds of crosslinked systems [25,27,28,32,34,69]. In our case, similar slopes were obtained for NR and IR samples, as expected, since polyisoprene is the main component of NR. On the contrary, other authors found significantly different linear relationships for series of IR and NR samples sulfur-cured in the same conditions, and attributed this behavior to microstructural differences between the two kinds of polymers, which lead to the different conformational statistics and stiffness levels of the polymer chains [34]. The non-zero intercept is due to the fact that all the elastically active constraints, i.e., both chemical crosslinks and physical entanglements, contribute to *D*_res_, while only chemical crosslinks and trapped entanglements that do not relax upon swelling contribute to *ν*_c,sw_ [25,27,32,34]. A decay of *D*_res_ toward zero has been theoretically predicted in the very high temperature limit and for crosslink densities approaching zero due to large-scale chain motions [70], which are too slow at the experimental temperature to produce observable effects. 

An NMR crosslink density can be defined as *ν*_c,NMR_ = 1/(2*M*_c,NMR_), where *M*_c,NMR_ can be calculated from *D*_res_ using Equation (6) [25]:

(6) Mc,NMR=2πdrefDres [kgmol]

This equation is derived under the assumption of Gaussian chain statistics for the end-to-end vector between constraints. Here, *d*_ref_ is a parameter that depends on the kind of polymer and on the theoretical model and approximations used to estimate the static-limit reference dipolar coupling (*D*_stat_/k) and chain stiffness. For all calculations of *ν*_c,NMR_, *d*_ref_ = 617 Hz was used, a value estimated from spin dynamics simulations for poly(*cis*-1,4-isoprene) [25]. The linear correlations between *ν*_c,NMR_ and *ν*_c,sw_ (Figure 4c) are characterized by slopes higher than 1 (1.6 for IR and 1.7 for NR), similar to those found by other authors for sulfur-cured IR and NR networks [25,27,32,34]. This result can be explained by considering that values of both NMR and swelling crosslink densities are model-dependent and can be affected by systematic errors. In particular, it was demonstrated that the affine model used to predict the elastic behavior of swollen rubbers leads to an underestimation of the crosslink density [27]. On the other hand, in the case of NMR, the presence of topological interactions between polymer chains can impose further restrictions to segmental motions leading to a higher local order, i.e., higher average *D*_res_, than that theoretically predicted. Furthermore, slope deviations from unity can also be due to a possible increase in the contribution of physical entanglements to *ν*_c,NMR_ upon crosslinking, as observed in the literature from the comparison of data obtained for bulk and swollen samples [34].

#### 3.2.2. ^1^H Transverse Relaxation by CPMG Experiments

In crosslinked rubbers, the ^1^H transverse magnetization decay measured by Hahn echo or CPMG experiments is mainly determined by the dephasing process due to the average residual dipolar interaction of the polymer network chains undergoing non-isotropic fluctuations between constraints; minor contributions arise from faster isotropic motions of dangling chains and possible sol components. While the latter contributions are typically described by exponential functions, different analytical functions have been proposed to describe the contributions from inter-crosslinks chains. In particular, theoretical models that combine the Bloembergen–Purcell–Pound (BBP) theory for fast motions and the Andersen–Weiss formula to take into account the residual “solid-like” behavior have been devised [24,26,50,51]. A Gaussian decay of transverse magnetization has been predicted in the short time limit, with a characteristic time constant inversely proportional to the residual dipolar interaction, *D*_res_. However, these functions contain a large number of intercorrelated parameters, which prevent *D*_res_ values from being reliably determined [53]. 

**Figure 5:**
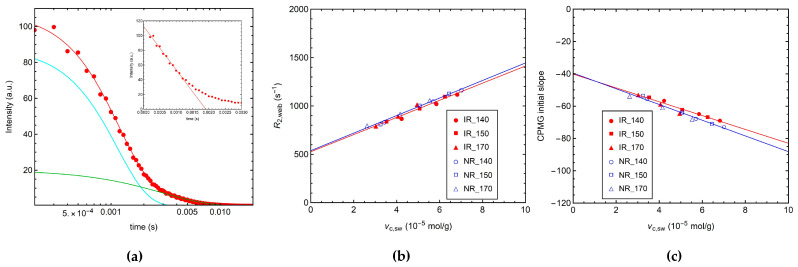
(**a**) Fitting of the ^1^H CPMG curve of IR_S1_140. Red dots represent the experimental data, while solid lines denote the analytical fitting function (red) and the single contributions of the Weibullian (cyano) and exponential (green) functions; in the inset, the same data are reported, with the solid line representing the linear fit to the first datapoints (up to 1 ms) of the CPMG curve. (**b**) ^1^H *R*_2,weib_ obtained from the fitting of the CPMG curves and (**c**) CPMG initial slope vs. *ν*_c,sw_. Solid lines represent the linear fits to the data.

In this work, we used a phenomenological approach to the analysis of the CPMG decay curves, which were reproduced using a linear combination of a Weibullian function and an exponential function (Equation (3)) [59,60,61], characterized by the decay constants *T*_2,weib_ and *T*_2,exp_, respectively. The Weibullian function represents the contribution of the network chains fluctuating between constraints responsible for the short time decay of transverse magnetization, while the long time decay, ascribable to dangling chains and sol components, is described by the exponential function. Figure 5a shows, as an example, the fitting of the CPMG decay curve of IR_S1_140, which allows a satisfactory reproduction of the experimental data. The best-fitting parameters obtained for all investigated samples are reported in Tables S1 and S2. In all cases, the Weibullian component accounts for the majority of protons in the sample (81–88%) and is characterized by *T*_2,weib_ values of ∼1 ms. The remaining fraction of protons gives the exponential decay with longer *T*_2,exp_ values of ∼3 ms.

As shown in Figure 5b, good linear relationships are found between the relaxation rate *R*_2,weib_ = 1/*T*_2,weib_ and *ν*_c,sw_ for all the samples. In particular, *R*_2,weib_ linearly increases by increasing *ν*_c,sw_, in agreement with the corresponding increase in *D*_res_ (Figure 4b). Similar slopes of about 90 s^−1^/(10^−5^ mol/g) with intercepts of about 530 s^−1^ are obtained for the IR and NR series.

Since we observed a good linear trend for the magnetization decay in the 0–1 ms time interval (see the inset of Figure 5a), the slope in this range (CPMG initial slope) was tested as a model-free parameter to be correlated with the crosslink density (see Section 2.5). As for *R*_2,weib_, good linear correlations with *ν*_c,sw_ were found for the CPMG initial slope (Figure 5c), with slopes of −4.3 and−4.9 s^−1^/(10^−5^ mol/g) and intercepts of −40 s^−1^ for IR and NR series, respectively.

#### 3.2.3. ^1^H Longitudinal Relaxation by FC NMR

^1^H longitudinal relaxation is driven by the fluctuations of magnetic dipole-dipole interactions between ^1^H-^1^H spin pairs under the effect of molecular motions. ^1^H FC relaxometry has proven to be particularly useful for the study of dynamics of uncured and crosslinked elastomers, since *R*_1_ depends on different power laws of the Larmor frequency in the different dynamic regimes governing relaxation [71,72]. It has been previously shown that the NMRD curves are dominated by glassy dynamics, and that on the basis of the FTS principle, it is possible to determine *τ*_s_ values from NMRD curves acquired at different temperatures [42].

**Figure 6:**
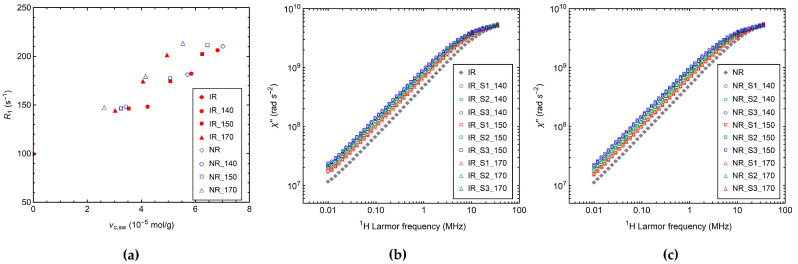
(**a**) ^1^H *R*_1_ values measured at the Larmor frequency of 0.19 MHz and 30 °C vs. *ν*_c,sw_. NMR susceptibility (*χ*″) curves obtained from the ^1^H NMRD curves of the (**b**) IR and (**c**) NR samples.

In this work, in order to explore the possibility to derive from *R*_1_ measurements a parameter to be correlated with the crosslink density through a fast procedure, we tested the values of ^1^H *R*_1_ measured at single temperature and Larmor frequency. In Figure 6a, *R*_1_ values measured at 0.19 MHz and 30 °C are reported as a function of *ν*_c,sw_. For samples vulcanized at the same *T*_vulc_, *R*_1_ progressively increases by increasing *ν*_c,sw_, with a steeper increase at the highest curing temperature of 170 °C. This trend, similar to that observed for *T*_g_, is ascribed to the fact that both parameters are affected by glassy dynamics, which is influenced not only by crosslinking but also by chemical modifications of the polymer chains.

**Figure 7:**
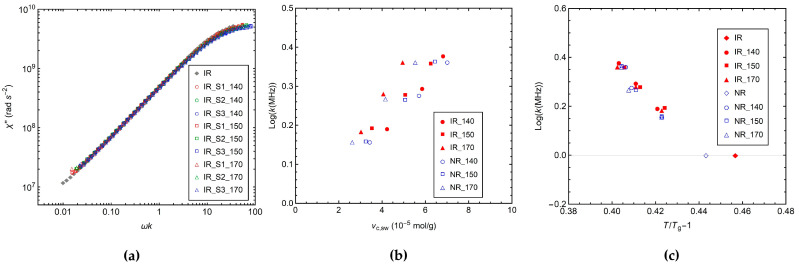
(**a**) NMR susceptibility (*χ*″) master curves of the IR samples obtained as described in Section 2.6. (**b**) Log*k* vs. *ν*_c,sw_ and (**c**) Log*k* vs. *T*/*T*_g_ − 1.

In order to investigate the effect of the vulcanization conditions on ^1^H *R*_1_ in more detail, it is useful to look at the whole NMR susceptibility (*χ*″) curves (Figure 6b,c) obtained from NMRD curves, as described in Section 2.6. A shift of the *χ*″ curves towards lower frequencies is observed, passing from the uncured to the crosslinked samples and by increasing the sulfur content, which is ascribable to the slowdown of glassy dynamics upon crosslinking [42]. ^1^H *χ*″ master curves of IR samples are shown as examples in Figure 7a, while those of NR samples are reported in Figure S2. As can be seen, after the application of the shift factor *k* along the frequency axis, the *χ*″ curves of the crosslinked samples perfectly overlap with that of the uncured one, indicating that neither crosslinking nor chemical modifications of the polymer chains affect the spectrum of motions, in agreement with the literature [39,40,41,42,73]. Different shift factors *k* are found for the different samples (Figure 7b); for each *T*_vulc_ the values of *k* progressively increase with the crosslink density, due to the slowdown of glassy dynamics induced by the formation of permanent constraints. In particular, the dependences of Log*k* on *ν*_c,sw_ and *T*_vulc_ reflect those observed for *R*_1_.

Under the assumption of a VFT dependence of *τ*_s_ on temperature (Equations (4) and (5)), the values of Log*k* are plotted vs. the reduced variable *T*/*T*_g_ − 1 (Figure 7c). As we can see, the curves for all samples from the same polymer overlap well, indicating that if *τ*_0_ and the fragility index *m* remain the same, the observed variations of *τ*_s_ should be mainly ascribed to changes of *T*_g_.

## 4. Conclusions

In this work, different TD-NMR methods—including measurements of ^1^H transverse and longitudinal relaxation times and of residual dipolar couplings, *D*_res_—were applied to the study of IR and NR samples vulcanized in different conditions. In particular, the effects of curing temperature and sulfur content were explored, while keeping constant the accelerator amount. The comparison of NMR results with data from equilibrium swelling and DSC experiments provided information on structural and dynamic features of the formed polymer networks and on their effects on the investigated NMR observables.

Chemical modifications of the polymer chains were found to occur at high curing temperatures, which hinder fast segmental motions, causing at the molecular level increased characteristic times of the glassy dynamics, *τ*_s_, as reflected by the longitudinal relaxation behavior investigated by ^1^H FC NMR, and causing at the macroscopic level an increased glass transition temperature, as measured by DSC. On the other hand, both ^1^H *D*_res_ and effective transverse relaxation rates showed good linear relationships with the crosslink density measured by swelling experiments, indicating a strong correlation of these NMR parameters with the formation of the rubber network but a substantial independence from the chemical modifications of polymer chains. The slopes obtained for IR and NR were similar, and in the case of *D*_res_ in agreement with those reported in the literature. The obtained results support the utility of the adopted TD-NMR methods for the characterization of the network structure of rubber materials at the molecular level. In particular, both *D*_res_ and *T*_2_ measurements were confirmed to be valuable empirical approaches to estimate the crosslink density of the polymer network, either as a complementary or even alternative approach to more widely used macroscopic methods, such as equilibrium swelling and mechanical measurements.

## Data Availability

The data presented in this study are available on request from the corresponding authors.

## Supplementary Materials

The following are available online at https://www.mdpi.com/article/10.3390/polym14040767/s1: Figure S1: Maximum torque (*M*_h_) determined by MDR measurements vs. *ν*_c,sw_. Table S1: Best fit parameters obtained from the analysis of the CPMG relaxation curves of the sulfur-cured IR samples. Table S2: Best fit parameters obtained from the analysis of the CPMG relaxation curves of the sulfur-cured NR samples. Figure S2: NMR susceptibility (*χ*″) master curves of the NR samples.
