# Supplementary material for: Influence of Sulfur-Curing Conditions on the Dynamics and Crosslinking of Rubber Networks: A Time-Domain NMR Study
Source: Polymers (Basel). 2022 Feb 16;14(4):767. doi: 10.3390/polym14040767 (PMC8880187; doi:10.3390/polym14040767)
Supplement: Supplementary file 1 [file polymers-14-00767-s001.zip › polymers-1591587-supplementary.pdf]

Influence of Sulfur-Curing Conditions on the Dynamics and Crosslinking of Rubber Networks: A Time-Domain NMR Study

Francesca Nardelli [1], Lucia Calucci [2,3,*], Elisa Carignani [2,3], Silvia Borsacchi [2,3], Mattia Cettolin [4], Marco Arimondi [4], Luca Giannini [4], Marco Geppi [1,3,*], and Francesca Martini [1,3,*]

1 Dipartimento di Chimica e Chimica Industriale, Università di Pisa, 56124 Pisa, Italy; francesca.nardelli@dcci.unipi.it (F.N.);
2 Istituto di Chimica dei Composti OrganoMetallici, Consiglio Nazionale delle Ricerche, 56124 Pisa, Italy; elisa.carignani@pi.iccom.cnr.it (E.C.); silvia.borsacchi@pi.iccom.cnr.it (S.B.);
3 Centro per l'Integrazione della Strumentazione Scientifica dell'Università di Pisa (CISUP), 56126 Pisa, Italy;
4 Pirelli Tyre SpA, 20126 Milano, Italy; mattia.cettolin@pirelli.com (M.C.); Marco.Arimondi@pirelli.com (M.A.); Luca.Giannini@pirelli.com (L.G.)
* Correspondence: lucia.calucci@pi.iccom.cnr.it (L.C.); francesca.martini@unipi.it (F.M.); marco.geppi@unipi.it (M.G.); tel: +39-0503152517 (L.C.); +39-0502219289 (M.G.); +39-0502219353 (F.M.)

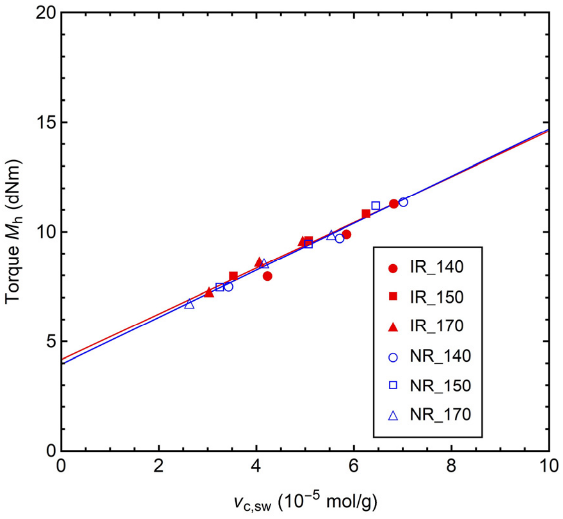

Figure S1. Maximum torque (M_h) determined by MDR measurements *vs* $\nu_{c,sw}$.

Table S1. Best fit parameters obtained from the analysis of the CPMG relaxation curves of the sulfur cured IR samples, performed as described in paragraph 2.5 of the main text.

Sample	$T_{2,weib}$ (ms)	C_{weib} (%)	β	$T_{2,exp}$ (ms)	C_{exp} (%)
IR_140_S1	1.15	81	1.5	2.95	19
IR_140_S2	0.98	84	1.5	3.34	16
IR_140_S3	0.89	82	1.5	2.76	18
IR_150_S1	1.20	84	1.5	2.95	16
IR_150_S2	1.03	86	1.4	2.77	14
IR_150_S3	0.92	81	1.5	2.64	19
IR_170_S1	1.26	84	1.5	2.92	16
IR_170_S2	1.11	84	1.4	2.76	16
IR_170_S3	0.98	83	1.4	2.69	17

Table S2. Best fit parameters obtained from the analysis of the CPMG relaxation curves of the sulfur cured NR samples, performed as described in paragraph 2.5 of the main text.

Sample	$T_{2,weib}$ (ms)	C_{weib} (%)	β	$T_{2,exp}$ (ms)	C_{exp} (%)
NR_140_S1	1.20	81	1.6	2.51	19
NR_140_S2	0.95	85	1.5	2.49	15
NR_140_S3	0.86	84	1.5	2.28	16
NR_150_S1	1.24	87	1.6	3.54	13
NR_150_S2	1.00	84	1.5	2.73	16
NR_150_S3	0.89	88	1.5	2.92	12
NR_170_S1	1.25	88	1.5	3.42	12
NR_170_S2	1.09	88	1.5	3.43	12
NR_170_S3	0.94	86	1.5	2.65	14

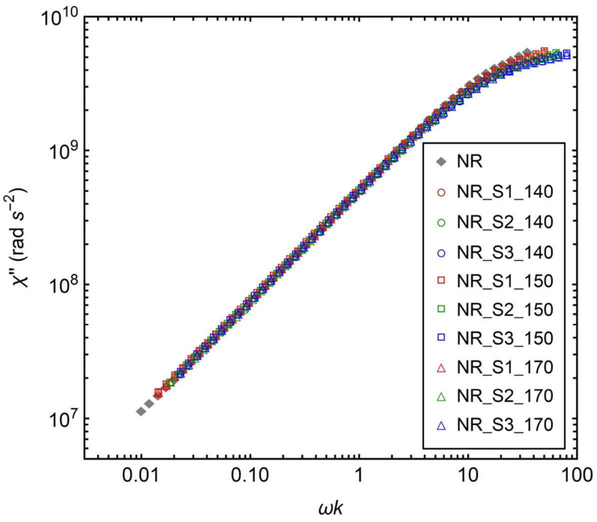

Figure S2. NMR susceptibility (χ'') master curves of the indicated NR samples obtained as described in paragraph 2.6 of the main text.
